# Testing Homeopathy in Mouse Emotional Response Models: Pooled Data Analysis of Two Series of Studies

**DOI:** 10.1155/2012/954374

**Published:** 2012-04-04

**Authors:** Paolo Bellavite, Anita Conforti, Marta Marzotto, Paolo Magnani, Mirko Cristofoletti, Debora Olioso, Maria Elisabetta Zanolin

**Affiliations:** ^1^Department of Pathology and Diagnostics, University of Verona, 37134 Verona, Italy; ^2^Department of Public Health and Community Medicine, University of Verona, 37134 Verona, Italy

## Abstract

Two previous investigations were performed to assess the activity of *Gelsemium sempervirens* (*Gelsemium s.*) in mice, using emotional response models. These two series are pooled and analysed here. *Gelsemium s.* in various homeopathic centesimal dilutions/dynamizations (4C, 5C, 7C, 9C, and 30C), a placebo (solvent vehicle), and the reference drugs diazepam (1 mg/kg body weight) or buspirone (5 mg/kg body weight) were delivered intraperitoneally to groups of albino CD1 mice, and their effects on animal behaviour were assessed by the light-dark (LD) choice test and the open-field (OF) exploration test. Up to 14 separate replications were carried out in fully blind and randomised conditions. Pooled analysis demonstrated highly significant effects of *Gelsemium s.* 5C, 7C, and 30C on the OF parameter “time spent in central area” and of *Gelsemium s.* 5C, 9C, and 30C on the LD parameters “time spent in lit area” and “number of light-dark transitions,” without any sedative action or adverse effects on locomotion. This pooled data analysis confirms and reinforces the evidence that *Gelsemium s.* regulates emotional responses and behaviour of laboratory mice in a nonlinear fashion with dilution/dynamization.

## 1. Introduction

*Gelsemium sempervirens* (Loganiaceae) is a twining vine containing the toxic strychnine-related alkaloids gelsemine, gelsemine, and sempervirine [1]. At pharmacological doses, *Gelsemium s.* has been reported to show sedative, analgesic, and antiseizure properties [2–5]. In homeopathic Materia Medica, *Gelsemium s. *is described as a remedy for a variety of anxiety-like neurological and behavioural symptoms [6–8], and there is a preliminary report [9] that homeopathic dilutions/dynamizations of *Gelsemium s.* in mice counter the effects of the anxiogenic compound RO 15-3505 (inverse agonist of benzodiazepines) in the labyrinth test. More recently, Bousta et al. have reported that, in some but not all experimental conditions, *Gelsemium s.* at the 5th, 9th, and 15th centesimal homeopathic dilutions/dynamizations (C) reduces stress-induced behavioural alterations of mice in the staircase test and light-dark test [10]. However, all these results represent reversals of the effects of severe stress (conditioned paradigm), and the findings vary widely depending on the dose administered and the test performed. There is therefore scope for further studies exploring the effects of *Gelsemium s.* in mouse models of emotional response, using rigorous methods.

Experimental investigations carried out with highly diluted solutions have suffered from problems of replicability between different laboratories [11–13] and even within the same laboratory using different experimental protocols [14]. To fill this gap, we performed two separate series of investigations [15, 16], using validated animal models, namely, the light-dark choice test (LD) and the open-field exploration test (OF), in order to acquire various behavioural parameters widely used in neuropsychopharmacology for drug screening [17]. In LD test, an increase in the amount of time spent in the lit compartment is an indicator of decreased anxiety, and the number of light-dark transitions has been reported to be an indicator of activity exploration over time. In OF, the total distance travelled in the arena reflects general exploratory activity, which may be altered by locomotor ability or motivational factors, and is reduced in case of sedation, paralysis, or impairment of movements, while the time spent and the distance travelled in centre reflect anxiolytic-like activity.

In the first paper [15], we describe the effects of *Gelsemium s.* 5C dilution/dynamization, followed by some exploratory tests using the 7C and 30C. In essence, it showed that, in the OF test, *Gelsemium s.* 5C, 7C, and 30C significantly increased the time spent and the distance travelled in the central zone. Neither dilution/dynamization of *Gelsemium s.* affected the total distance travelled, indicating that the behavioural effect was not due to unspecific changes in locomotor activity. In the LD test, *Gelsemium s.* 5C and also 30C showed a positive effect in the same direction as diazepam but did not reach the statistical significance. In the light of these partially positive results, we decided to continue and intensify our study and undertook a new experimental series with 6 replications of a similar protocol where a wider range of *Gelsemium s.* dilutions/dynamizations (4C, 5C, 7C, 9C, 30C) was tested. Minor protocol differences concerned the sequence of testing, the housing of animals, and the supplier of mice (see Methods). A preliminary report of the results of the second series appeared in a review [18], and the complete study was published in Psychopharmacology [16]. *Gelsemium s.* 5C, 7C, 9C, and 30C showed stimulatory activity on the time spent and distance travelled in the central zone of the OF, but this effect did not go beyond the threshold of statistical significance (*P* = 0.060). On the other hand, with the LD test parameters, in the second series, the effect of *Gelsemium s.* was much more evident and significant (*P* < 0.01 in the global ANOVA for groups): the medicament at the 5C, 9C, and 30C dilutions/dynamizations increased the time spent in the light compartment (by 21.58%, 37.47%, and 21.85%, resp.) and the number of transitions (by 24.66%, 40.01%, and 40.02%, resp.), with high statistical significance. These effects were in the same direction as those of diazepam and buspirone, tending to confirm an anxiolytic-like activity. In summary, these two series of studies yielded qualitatively similar results, but with notable quantitative variations. Others have recently raised the issue of reproducibility of *Gelsemium s. *effects [19]. Therefore, in order to verify whether the effects on the considered behavioural variables are consistent, significant, and reproducible, we present here a complete summary of these investigations, with a new analysis of pooled data.

## 2. Methods

### 2.1. Animals

All the experiments were performed at the Faculty of Medicine, Verona University, Italy, with some minor modifications between the two series of replications (Table 1). Male mice 4-5 weeks old of the CD1 strain were purchased from Harlan Laboratories (Udine, I) or Charles River Laboratories (Lecco, I) and allowed to acclimate for two weeks before testing, in a controlled animal facility (temperature 22 ± 2°C, humidity 55% ± 5%). The mice were randomly distributed, 4 per cage (size 349 × 156 × 132 mm) or 2 per cage (size: 250 × 140 × 120 mm) in plastic cages, and housed with food and water available ad libitum, except for during the brief testing periods. Lights were on between 7 AM and 7 PM.

In each replication, groups of mice (*n* = minimum 8, maximum 16) were randomly assigned to separate cages and treated with different solutions as indicated. The arrangement of cages in the laboratory rack and the order in which mice were injected and tested were evenly distributed for all cages and experimental groups, to avoid cage effects and other possible biases linked to the timing of injections and tests. Each animal was used only once in the same test to avoid the confounding effects of learning and habituation. Each replication experiment lasted about 4 weeks, including animal habituation, drug delivery, testing, and data collection and analysis.

### 2.2. Drugs and Treatments

The drugs were produced by Boiron Laboratoires, Lyon (F), starting from a crude hydroalcoholic extract of fresh underground portions of *Gelsemium s.*, which was diluted 100 times in 30% ethanol/distilled water to obtain the 1C dilution/dynamization. Subsequent serial 100 × dilutions followed by vigorous shaking (dynamization) of up to 29C were then made in the same solvent, using glass bottles. The content of gelsemine—the principal alkaloid of *Gelsemium s.*—in the first hydroalcoholic extract was 0.021% (w/v), corresponding to a concentration of 6.5 × 10^−4^ moles/L. The control solution (vehicle) was the same batch of 30% ethanol/distilled water solution used to prepare the drug samples.

The solutions were stored in the dark at room temperature in an aluminium envelope. Before being used in each replication experiment, 0.4-mL samples of each solution (including the control solution) were added to 39.6 mL of distilled sterile and apyrogenic water in a sterile 50-mL Falcon plastic tube, closed with a plastic cap, and manually shaken with 20 strong vertical strokes to obtain the final drug samples and control vehicle used for treatments, with final ethanol concentration lowered to 0.3% (v/v). Diazepam (Valium, Roche, final dose of 1 mg/kg) or buspirone (Sigma, final dose of 5 mg/kg) were diluted in the final vehicle solution (0.3% ethanol in distilled water). Preliminary experiments, comparing a 0.3% ethanol solution in distilled water (final dose 0.03 g/kg) with pure distilled water, showed that this dose of diluted ethanol does not affect behaviour of mice in any of the test employed. In order to blind the operators with respect to the test solutions, all the samples were then coded by an independent person and the codes kept sealed inside an envelope until all the tests and calculations were completed. The solutions were distributed in 15-mL sterile Falcon plastic tubes (4 mL/tube), wrapped in aluminium foil, and stored at +4°C until the day of use. Before being used, each tube was again manually shaken with 20 strokes. All the procedures were performed in sterile conditions and using sterile disposable plasticware.

The drug and control solutions were administered in the morning for 9 consecutive days (including on the last two days, when the behavioural tests were carried out) by intraperitoneal (i.p.) injection (0.3 mL) using disposable 1-mL (insulin) syringes. The diazepam-treated group received the drug solution only on the days of testing, in consideration of the well-known development of tolerance to benzodiazepines [20] and their short half-life [21], and 0.3 mL of control solvent solution (0.3% ethanol/distilled water) for the first 7 days. The treatment and testing procedures were independently approved by the Animal Ethics Committee of the Interdepartmental Centre for Animal Research (CIRSAL) of Verona University and by the Italian Health Ministry. Aside from the treatment injections and testing, the animals were not subjected to pain or other forms of emotional or physical stress.

### 2.3. Behavioural Tests

**Figure 1:**
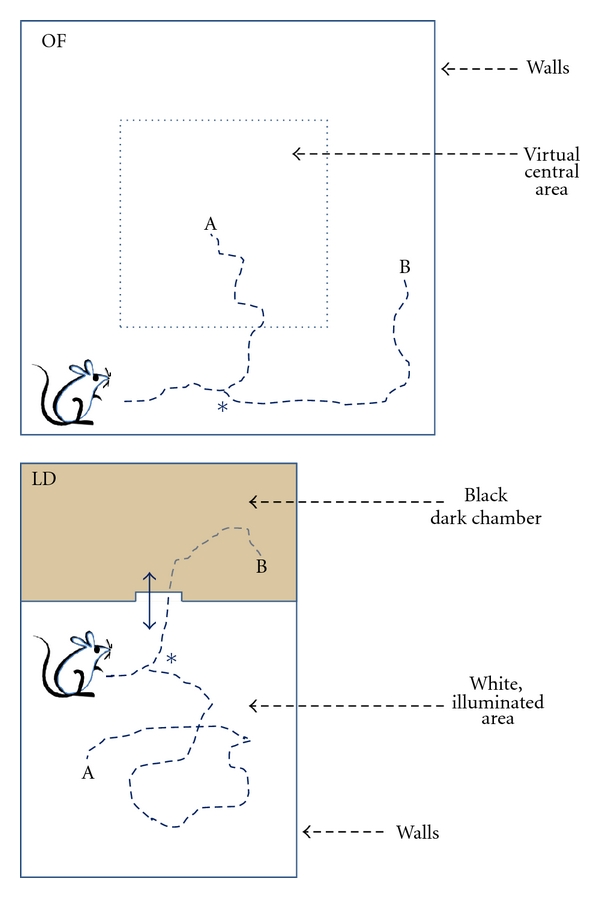
Schematic representation of the arenas of the OF (above) and LD (bottom) tests. The hypothetical bifurcation point of the trajectory choice is indicated by an asterisk. (A) positive effect of the drug (less anxiety, less fear, more exploration attitude), (B) negative effect of the drug.

The OF behaviour test (Figure 1, above) [22–24] involves placing an animal in an unfamiliar environment consisting of a 50 × 50 cm black-painted wooden platform, with 25 cm high surrounding walls, illuminated with white light (100 lux). The OF arena is virtually divided into two parts, with a square central zone having an area corresponding to 25% of the total area. The percentage time spent in this central zone is considered indicative of exploratory behaviour and may reflect a decrease in anxiety, although this OF parameter is not sensitive to all anxiolytics and may not model certain features of anxiety disorders [24].

The LD exploration test (Figure 1, bottom) [25–27] is based on the innate aversion of rodents to brightly lit areas, and their spontaneous exploratory behaviour in response to mild stressors such as novel environments and light. Mice tend to prefer dark, enclosed spaces to large, well-lit areas, and the amount of time spent in the dark zone is sensitive to benzodiazepines and to the agonists of serotoninergic receptors, in a manner that correlates well with clinical efficacy in humans [28]. The test apparatus consists of a small, secure dark compartment (15 × 30 cm), and a large, aversive illuminated compartment (30 × 30 cm). The two compartments are separated by a partition with an opening (4 × 4 cm) through which the animal can pass from one compartment to the other. The open arena is brightly illuminated with 200 lux, and the mice are left to explore the space for a 5 min testing period. The score for the transition was assigned, by a person not aware of the treatment assignment of the groups, from the analysis of the video recordings, when the animal came out of the dark chamber with all 4 paws.

The animals were tested individually in 4 separate devices, starting from 30 min after last drug (or placebo) administration. In the first series of replications [15], the treatments were delivered before all test procedures, and the assays performed in the following order: LD on 8th day of solution administration, OF on the following day (9th day of solution administration). Since the best results were obtained in the OF (see results), in the second series [16], the assays were performed in the following order: OF on 8th day of solution administration, and LD on the following day (9th day of solution administration). To match better the timing of testing with that of drug administration, in the second series of replications, the drugs were delivered row by row (8 cages of 2 mice in each row of the housing rack), followed by testing of the injected animals, so that the test procedures were completed 80–90 min after the last treatment. Immediately before testing, the animals were allowed to acclimate to the room inside their cages for three minutes, after being brought there from their customary housing area. The operators stayed outside the testing room during the recording of the experimental sessions. In very few cases, a mouse was lost because it jumped out of the test arena during the test, and those cases were excluded from the calculations. The test arenas were cleaned thoroughly with water and soft disposable paper between trials and with water and detergent between experiments.

### 2.4. Image Analysis System

A video-tracking camera and software program (“Smart” VTS system from PanLab, Barcelona, E) were used to record the sessions automatically. Essentially, this system consists of a video-camera (GZ-MG135, JVC, Japan) mounted on the ceiling 2.5 m above the centre of the experimental field, a video interface, and a computer. The camera views 4 test arenas, each of which is in turn divided by the software into two zones, depending on the test to be performed (LD or OF). All the sessions are recorded and stored on DVDs. The acquired video signals are converted by the image processor into binary images in which the animal appears as a black spot against a white background. The movements of the spot are recorded to track the animals' position, the amount of time spent in different zones, and the distance travelled.

### 2.5. Statistics

Up to 14 replications from two series of studies were performed and analysed: 8 replications of the same protocol in the first series [15], and 6 replications in the second series [16]. Analyses were performed using the Stata12 software (http://www.stata.com) and the SPSS 17 software (http://www.spss.com). The effect of the drugs on each mouse was calculated as a percentage relative to the mean values for the controls (vehicle-treated) in each replication of the series, taken as zero effect, according to the formula:

(1) $$\begin{array}{cc} & \left[\left(\frac{\text{Test value of each mouse}}{\text{mean test value of control mice}}\right)-1\right]\times 100. \end{array}$$

This standardisation allowed the effects observed in all the experiments to be compared and statistically evaluated. All data are represented as mean ± SEM (standard error of the mean) values. The pooled data were normally distributed. Nested ANOVA was used to find any differences in the studied parameters (time spent and distance travelled in the centre of OF, total distance travelled in OF, time spent in the lit area of LD box, number of dark-light transitions) according to type of treatment and controlling for experimental series, and replications (with replications nested in experimental series). When global ANOVA was significant and there was no interaction between groups and series, the data of the two series were pooled and specific comparisons were assessed to determine differences between groups. Post hoc *t*-tests were performed assuming equal variances with least significant difference corrections to adjust for multiple comparisons (protected LSD), as suggested by a consensus report [29] for basic research in high dilution/dynamization pharmacology. Pearson correlation coefficient (*r*) was used to analyse the association between different behavioural variables in the control groups.

## 3. Results

### 3.1. Open Field

The results of pooling all the tests performed with OF test is reported in Table 2. An interaction between series and groups emerged only for the variable “distance in centre” in OF that was therefore excluded from subsequent analysis.

In the variable “time spent in centre,” a difference that did not reach statistical significance was noted in global ANOVA for series. However, there was no interaction between series and groups, indicating that the drug effects were in the same direction in all groups, albeit with quantitative differences.

There were highly significant differences between groups. All *Gelsemium s.* samples except for 4C showed a stimulatory activity as compared with control solvent, with a statistically significant difference for the 5C, 7C, and 30C dilutions/dynamizations. Equally apparent is the lack of effect of the two standard drugs diazepam and buspirone on these parameters, suggesting that this model system in these experimental conditions was not suitable for detecting a conventional anxiolytic effect and hence that the effect of *Gelsemium s.* on mouse behaviour in the OF is qualitatively different from that of standard drugs (see also Discussion).

During the OF test, the total distance travelled by the mice in the arena was also analysed (Table 2). Considering the entire series of replications, no significant differences were found between various groups, although a small inhibitory effect was found in buspirone-treated versus solvent-treated animals (−9.19%), suggesting a possible sedative effect instead of anxiolytic-like effect. This phenomenon was not present with diazepam and *Gelsemium s.*, suggesting that these drugs did not affect the unspecific locomotor activity of the mice and the observed differences in time spent in the central zone were due to genuine changes of anxiety levels.

### 3.2. LD Test

As shown in Table 3, the time spent in the open, illuminated (white) compartment of the LD test arena increased in all the *Gelsemium s.*-treated groups and in the groups treated with diazepam and buspirone. Considering the whole of this large population of animals, the effects of *Gelsemium s.* C5, C9, and C30 proved highly statistically significant in post hoc analysis, with a peak at 9C dilution/dynamization. Similar results were obtained by measuring the number of transitions between compartments, with the difference, as compared with the permanence time, that here only the effects of 9C and 30C dilutions/dynamizations proved to be statistically significant. Moreover, in this test, parameter buspirone was less effective as positive control. Since this parameter is likewise linked to physical motility, this may be due to the slight inhibitory effect of buspirone on unspecific locomotion already noted in OF. In LD responses, there were no significant differences in the effects in the two series.

### 3.3. Differences between Behavioural Parameters

**Figure 2:**
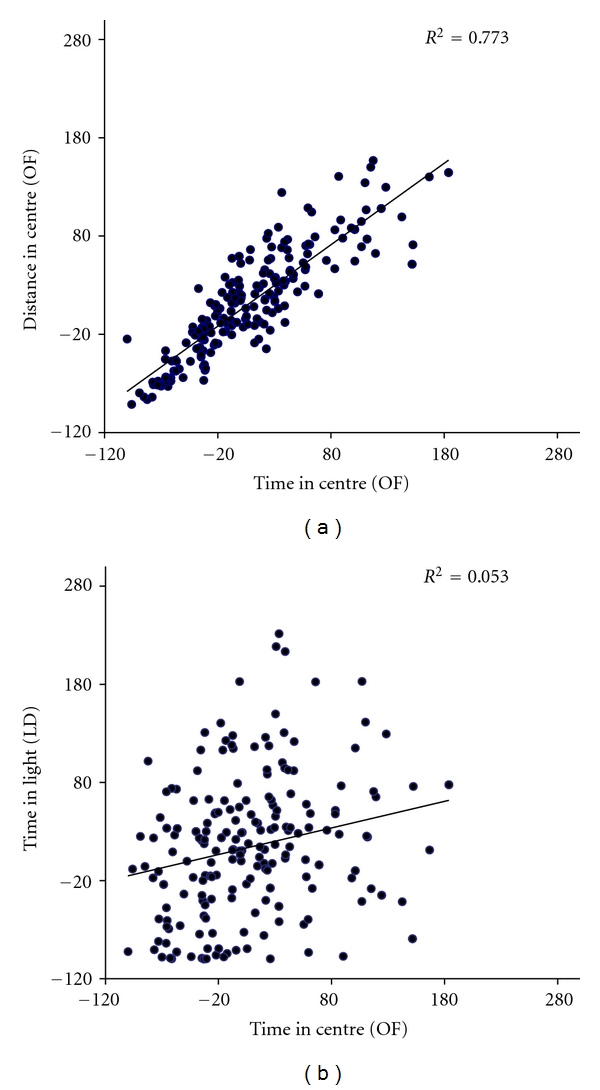
Correlations (Pearson's *r*) between different behavioural variables explored by the OF and LD tests. The data points for mice from the control groups of all replications were utilised.

The effects of *Gelsemium s.* displayed marked nonlinearity with dilution/dynamization and were different in the OF and LD assessments. In the OF, the 4C was inactive and showed significantly lower effects than the 5C, 7C, and 30C. In the LD, the activity of the 7C dilution/dynamization was very low, while peak activation was noted using the 9C. In the OF test, there was a significant effect of *Gelsemium s.* (peak 7C) but not of the conventional drugs, while, in the LD test, both *Gelsemium s.* (peak 9C) and the conventional drugs showed significant effects. These discrepancies strongly suggest that the two test paradigms explore different behavioural symptoms which respond differently to conventional and homeopathic drugs. This conclusion is supported by the finding illustrated in Figure 2. Utilising all the data points for untreated control mice, we observe a clear relation between the two OF parameters (time spent and distance travelled in the centre), indicating that both reflect a decision of whether to stay in the peripheral area (thigmotaxis) or to explore the central area. On the other hand, the time spent in the centre of the OF does not correlate with the time spent in the lit area of the LD, suggesting that these two parameters reflect different physiological features and behavioural parameters, and this may be the reason for the differing sensitivity to the treatments.

## 4. Discussion

Natural remedies are frequently used by people suffering from anxiety disorders, but evidence of their benefits in randomised controlled studies [30, 31] and laboratory research [18] is limited. Due to the controversial nature of homeopathic claims, it is important for any results in this field to be confirmed and consolidated through further investigations and rigorous statistical evaluation. Two previous investigations [15, 16] suggested that *Gelsemium s.* reduced anxiety and fear and increased exploratory behaviour in the laboratory mouse, without provoking any sedation side-effects. However, in the first series, the major and most significant effect was noted in OF parameters [15], while, in the second one, the LD test yielded the best results [16]. Since reproducibility, the degree of accordance between the results of experiments testing the same hypothesis, is a fundamental requisite for acceptance of any evidence, we performed a new analysis to evaluate statistically the differences between the two series and the global significance of the results.

In all parameters considered but one (distance travelled in centre in OF), there were no significant differences between the two experimental series nor interaction between series and experimental groups. This indicates that the trends of the drug effects were qualitatively in the same direction, despite a noteworthy quantitative variability. The pooled data analysis confirms and reinforces the evidence that statistically high significant *Gelsemium s.* effects can be detected in the laboratory mouse using both the OF and LD paradigms, even with the high dilutions/dynamizations employed in the homeopathic pharmacopoeia (9C and 30C). This laboratory evidence, based on blinded protocols and using groups of large sample size, strongly supports the conclusion that homeopathic medicaments are not mere placebos and are endowed with specific pharmacological activity.

The ability of extremely diluted drugs to change these emotional responses of mice can be ascribed to the high sensitivity of the tests involved, which are designed to put the animal in a situation of uncertainty (“bifurcation point,” indicated by an asterisk in Figure 1), where an extremely slight influence can determine the choice of which direction to move in (A or B in figure). The sensitivity of these tests to minimal factors is also, conceivably, one reason for the high variability of responses in the two series of experiments, observed in both vehicle-treated and drug-treated animals. It has been noted that the extent to which an anxiolytic compound facilitates exploratory activity depends on its baseline level in the control group [25]. Bousta et al. [10] report some anxiolytic-like effects of *G. sempervirens* in mice stressed by repeated electric shocks, but no such effects in normal unstressed mice. Differences between the nature and severity of external stressors, or between experimental setups, environment, handling and testing, and individual biological responses to drugs, might account for the high variability of results reported under different experimental conditions [24, 32, 33]. Variable behavioural baseline levels have been reported by others [17, 34], and it has been found that two groups having low and high “trait” anxiety and different neuroendocrine responses to stress can be selected from the same mouse population [35], indicating that expression of trait anxiety displays a high interindividual variability in inbred mice.

In the OF model,* Gelsemium s.*-treated mice were unaffected in their general movement and locomotion in the field but showed a higher tendency to enter the central zone, instead of running along the walls or staying in the corners. This behaviour is thought to reflect changes in the emotional state of the mouse, even though in our experimental conditions, the OF parameters do not measure “anxiety” but rather exploratory propensity, thigmotaxis, and neophobia. This conclusion is based on the fact that neither buspirone nor diazepam altered those parameters. The differences between the effects of *Gelsemium s.* and those of the conventional anxiolytic drugs diazepam and buspirone suggest that the former has a broader action on animal behaviour, possibly including the stimulation of exploratory behaviour in the OF. The LD, on the other hand, proved to be a very valid test for anxiety, given that it always showed some effect with the two conventional anxiolytics, as well as with *Gelsemium s.*

Anxiety, neophobia, fear, and thigmotaxis are rather complex phenomena. There are two types of anxiety, “state” anxiety (excess anxiety experienced by a subject at a particular time in presence of a stimulus) and “trait” anxiety (does not vary from moment to moment) [36]. It has been suggested that the light-dark test and elevated plus-maze device are the most appropriate models for assessing state anxiety, while the free-exploratory paradigm can be used for “trait anxiety” [33, 37]. It has also been reported that anxiolytic treatments do not by themselves increase exploration in the central zone of the OF, but they do decrease the stress-induced inhibition of exploratory behaviour [17]. Benzodiazepines have been found to be inactive in some models or even to produce paradoxical anxiogenic effects [38]. That OF is less sensitive to benzodiazepines, and buspirone as compared with other behavioural tests, (e.g., elevated plus-maze) has been shown also by others [39], and a decrease of locomotion caused by buspirone at low (1 mg/kg) and high (10 mg/kg) doses has been observed in rats [40]. Further studies with additional tests of anxiety are needed to confirm this intriguing relationship.

These findings strongly suggest that the LD and OF tests explore different emotional responses, with different sensitivities to drugs and neurological mechanisms. Our data showing lack of correlation between responses with two test used (Figure 2) seem in agreement with this conclusion. In this connection, it is also worth noting that the peak of *Gelsemium s.* activity in the LD test was observed with the 9C dilution dynamization, while, in the OF, it occurred with the 7C. This may suggest that the different behavioural “symptoms” exploited by these two test paradigms are sensitive to different dilutions/dynamizations of the remedy.

A possible action mechanism of *Gelsemium s.* at neurological level has been indicated by others, showing that, in rat brain slices, very low doses [41] and high dilutions/dynamizations (5C, 9C) of this compound [42] enhance the production of the neurosteroid allopregnanolone (5a,3a-tetrahydroprogesterone), a stimulator of GABAa receptors and of inhibitory signalling in the central nervous system. These authors [41] showed that this activity was stimulated by glycine and blocked by strychnine, well known as a glycine receptor (Gly-R) antagonist, suggesting that gelsemium effects are antagonistic to those of strychnine and mediated by Gly-R receptors.

*Gelsemium s.* is frequently used in homeopathy to treat patients exhibiting neurological anxiety-like symptoms such as “general prostration, trembling, tired feeling, mental apathy, muscular weakness, complete relaxation and prostration, lack of muscular co-ordination, general depression, emotional excitement, bad effects from fright, fear, exciting news” according to the Materia Medica [6–8]. The fact that the traditional indications for the remedy are consistent with significant laboratory findings using rigorous experimental models helps bridge a gap between two medical disciplines generally considered to be at variance with each other, but which should instead be regarded as complementary and compatible. Of course, further scientific evidence of possible clinical benefits of homeopathy in humans is needed.

## Figures and Tables

**Table 1:** Features of the two series of experiments testing *Gelsemium s.* on mouse behaviour.

		Series no. 1	Series no. 2
Mouse producer		Harlan	Charles River
Housing		4/cage	2/cage
Light/dark cycle		Light during day (h 7–19)	Light during day (h 7–19)
Drug administration		0.3 mL/day for 8 days, i.p.	0.3 mL/day for 8 days, i.p.
Test sequence		1st day: light-dark 2nd day: open field	1st day: open field 2nd day: light-dark
Test schedule		30 min–320 min after last drug administration	30 min–90 min after last injection
Number of complete experiments	Control (solvent)	8	6
	Diazepam	5	1
	Buspirone	n.t.	5
	Gels 4C	n.t.	6
	Gels 5C	8	6
	Gels 7C	3	6
	Gels 9C	n.t.	6
	Gels 30C	2	6

**Table 2:** Cumulative results of open-field test (14 replications in two experimental series^§^).

Tested variable	Tested samples	Number of mice	Effect (% of control)		ANOVA		Post hoc test
			Mean	SEM	For series	For groups	
Time in centre	Control (solvent)	212	0.00	3.15	*F* = 4.44 *P* = 0.057	*F* = 4.72*P* < 0.0001	—
	Diazepam (1 mg/kg)	77	−1.20	6.96			0.867
	Buspirone (5 mg/kg)	40	8.48	8.02			0.362
	Gels 4C	48	−4.35	6.43			0.614
	Gels 5C	166	19.67	4.48			<0.001
	Gels 7C	95	29.80	6.12			<0.0001
	Gels 9C	48	13.82	7.05			0.110
	Gels 30C	80	26.29	7.09			<0.001

Total distance	Control (solvent)	213	0.00	1.52	*F* = 0.00 *P* = 0.951	*F* = 1.90*P* = 0.066	—
	Diazepam (1 mg/kg)	79	3.56	3.52			
	Buspirone (5 mg/kg)	40	−9.19	2.43			
	Gels 4C	48	2.45	3.03			
	Gels 5C	166	4.94	1.55			
	Gels 7C	94	3.45	2.04			
	Gels 9C	48	0.77	3.23			
	Gels 30C	79	4.96	2.54			

^§^For series definition, see Table 1.

**Table 3:** Cumulative results of light-dark test (14 replications in two experimental series^§^).

Tested variable	Tested samples	Number of mice	Effect (% of control)		ANOVA		Post hoc test
			Mean	SEM	For series	For groups	
Time in lit area	Control (solvent)	215	0.00	3.95	*F* = 6.31*P* = 0.24	*F* = 3.60*P* < 0.001	—
	Diazepam (1 mg/kg)	77	34.85	8.93			<0.0001
	Buspirone (5 mg/kg)	40	25.81	6.82			0.015
	Gels 4C	48	18.15	8.47			0.066
	Gels 5C	165	14.94	5.56			0.019
	Gels 7C	95	6.71	5.73			0.377
	Gels 9C	48	37.47	7.04			<0.001
	Gels 30C	79	16.15	6.27			0.047

Light/dark transitions	Control (solvent)	213	0.00	4.39	*F* = 7.69*P* = 0.09	*F* = 9.42*P* < 0.0001	—
	Diazepam (1 mg/kg)	78	86.78	15.70			<0.0001
	Buspirone (5 mg/kg)	40	11.88	6.60			0.386
	Gels 4C	48	21.78	11.43			0.086
	Gels 5C	165	16.09	6.87			0.051
	Gels 7C	95	7.22	5.60			0.461
	Gels 9C	48	40.01	9.09			0.002
	Gels 30C	79	33.12	7.43			0.002

^§^For series definition, see Table 1.
